# Hypervirulent *Mycobacterium tuberculosis* strain triggers necrotic lung pathology associated with enhanced recruitment of neutrophils in resistant C57BL/6 mice

**DOI:** 10.1371/journal.pone.0173715

**Published:** 2017-03-17

**Authors:** Fabrício M. Almeida, Thatiana L. B. Ventura, Eduardo P. Amaral, Simone C. M. Ribeiro, Sanderson D. Calixto, Marcelle R. Manhães, Andreza L. Rezende, Giliane S. Souzal, Igor S. de Carvalho, Elisangela C. Silva, Juliana Azevedo da Silva, Eulógio C. Q. Carvalho, Afranio L. Kritski, Elena B. Lasunskaia

**Affiliations:** 1 Laboratory of Biology of Recognition, Universidade Estadual do Norte Fluminense, Campos, Rio de Janeiro, Brazil; 2 Departament of Immunology, Biomedical Science Institute (ICB), University of Sao Paulo, Sao Paulo, Brazil; 3 Molecular MIcobacteriology Laboratory, Medicine School, Clementino Fraga Filho University Hospital, Federal University of Rio de Janeiro, Rio de Janeiro, Brazil; 4 Laboratory of Animal Morphology and Pathology, Universidade Estadual do Norte Fluminense, Campos, Rio de Janeiro, Brazil; 5 Tuberculosis Academic Program, Federal University of Rio de Janeiro, Rio de Janeiro, Brazil; Rutgers Biomedical and Health Sciences, UNITED STATES

## Abstract

Tuberculosis (TB) is a chronic infectious disease caused by *Mycobacterium tuberculosis* (Mtb) that in most cases induces irreversible necrosis of lung tissue as a result of excessive inflammatory reactions. The murine model of TB in resistant C57BL/6 mice infected with reference Mtb strains is widely used in TB studies; however, these mice do not show a necrotic pathology, which restricts their use in studies of irreversible tissue damage. Recently, we demonstrated that necrotic lung lesions could be induced in the C57BL/6 mice by highly virulent Mtb strains belonging to the modern Beijing sublineage. However, the pathogenic mechanisms leading to necrosis in this model were not elucidated. In this study, we investigated the dynamics of lung lesions in mice infected with highly virulent Beijing Mtb strain M299, compared with those infected with laboratory Mtb strain H37Rv. The data demonstrate that necrotic lung lesions in mice infected by the strain M299 were associated with enhanced recruitment of myeloid cells, especially neutrophils, and increased levels of proinflammatory cytokines, consistent with exacerbated inflammation. High levels of IFN-γ production contributed to the control of bacterial growth. Further progression to chronic disease was associated with a reduction in the levels of inflammatory mediators in the lungs, the accumulation of foamy macrophages and partial healing of the necrotic tissue by fibrosis. At a late stage of disease, degradation of foamy cells resulted in the liberation of accumulated lipids and persisting bacilli and further activation of inflammation, which promoted lung consolidation. Overall, our studies show that C57BL/6 mice infected with highly virulent Mtb strain may serve as a TB model reproducing an exacerbated inflammatory response in a resistant host to hypervirulent mycobacteria, leading to irreversible necrotic lung lesions.

## Introduction

Necrotic lesions are hallmarks of pulmonary tuberculosis (TB) pathology, including intragranulomatous necrosis, tuberculous pneumonia, pleurisy and extensive necrosis of post-primary TB lesions leading to cavitation. Necrosis contributes to the loss of lung function and provides a secure niche for bacteria, characterized by limited penetration of leukocytes and antibiotics into necrotic tissues [1]. The generation of new host-directed therapeutic approaches aimed at prevention or reduction of pulmonary necrosis as well as generation of anti-mycobacterial drugs able to penetrate necrotic lesions are important goals for research in TB treatment. Further progress in investigation of necrotic TB lesions depends on animal models that reproduce different types of the necrotic pathology. The major disadvantage of conventional murine models of TB based on TB-resistant lineages of mice, such as the C57BL/6 lineage, is the absence of necrotic pathology in these animals during infection with reference Mtb strains [2].

Several alternative murine models that reproduce necrotic TB lesions have been proposed. These models are based on mice exhibiting increased susceptibility to TB as a result of the selection of animals bearing genetic polymorphisms reducing natural immunity (lineages C3HeB/FeJ, DBA/2 or CBA/J), genetic modifications to disrupt key genes associated with host resistance (IFN-γ-, TNF-α- or iNOS- deficient mice), or treatment with TLR- agonists to strengthen the inflammatory response [3–9]. In most of these models, the development of necrotic lung lesions is associated with exacerbated uncontrolled inflammation driven by prominent neutrophilic influx into the lung, resulting in rapid pulmonary consolidation and early animal death, thereby reducing the utility of these models for drug trials. Few susceptible murine models are able to reproduce chronic disease accompanied by the development of encapsulated intragranulomatous necrotic lesions, such as the Type I lesions in C3HeB/FeJ mice. following infection with a low dose of bacilli [9]. However, around 30% of these mice still succumb to pre-mature death, mainly attributed to poorly organized necrotizing pneumonia (Type II lesions).

In our previously published studies, we described the course of TB in resistant C57BL/6 mice intratracheally infected with a low dose of highly virulent Beijing Mtb strains, leading to the development of multifocal necrotizing pneumonia [10,11], resembling the Type II lesions observed in C3HeB/FeJ mice [9]. However, the survival rate of C57BL/6 mice was significantly longer (median survival time of 180–200 d) than that of C3HeB/FeJ mice exhibiting similar pathological signs (28–45 d). For a better understanding of the pathogenesis of lung necrosis in the C57BL/6 model, we evaluated in this study the dynamics of pathological lesions over time, addressing cellular composition, cytokine production and mycobacterial load.

The data demonstrate that necrotic lesions in C57BL/6 mice infected with a highly virulent Mtb strain were associated with enhanced neutrophil influx and increased levels of inflammatory cytokines production in the lungs. However, in contrast to susceptible lineages, C57BL/6 mice were able to better control bacterial growth and lung inflammation, thus exhibiting transition to chronic disease. These findings link the pathological patterns of pulmonary necrosis in C57BL/6 mice to those observed in other experimental models and humans with severe forms of TB. Moreover, we suggest that this model could be useful for testing new approaches concerning the inhibition of hyperinflammatory host reactions, the reduction or prevention of pulmonary necrosis, as well as the elimination of extracellular mycobacteria persisting in necrotic lesions.

## Materials and methods

### Mycobacteria

The *M*. *tuberculosis* strain of the Beijing genotype (strain M299), isolated from a TB patient in the Maputo province, Mozambique, and the laboratory *M*. *tuberculosis* strain H37Rv (ATCC) were kindly provided by Dr. Philip Suffys (Oswaldo Cruz Foundation, FIOCRUZ, Rio de Janeiro, Brazil). The strains were obtained as Lowenstein-Jensen slants of low passage number cultures. Bacterial colonies were scraped and suspended in Middlebrook 7H9 broth (Difco, Detroit, MI), containing 10% albumin-dextrose-catalase complex, ADC (BD, Sparks, MD). Heavy suspensions of the microorganism were aliquoted and stored at − 80°C. Before experiments, the aliquots were thawed, resuspended in complete 7H9 medium, supplemented with ADC and 0.05% Tween-80, and cultured for 7 days at 37°C. Bacterial suspensions were ultrasonicated in water bath and mixed by vortex to disrupt small clumps and obtain single cell suspensions. The densities of the suspensions were measured by spectrophotometry, and corresponding concentrations were determined by the colony-forming units (CFU) test, using serial 10-fold dilutions of each strain suspension and plating on Middlebrook 7H10 agar (Difco, Detroit, MI), supplemented with 0.5% glycerol, 10% oleic acid–albumin-dextrose–catalase enrichment, OADC (BD, Sparks, MD), to establish viable bacterial cell count. After 21 days, total CFU were determined by counting.

### Mouse infections

Specific-pathogen-free C57BL/6 mice were originally purchased from the Biotério do Instituto de Ciências Biomédicas at the University of Sao Paulo (USP) and further bred and maintained under pathogen-free conditions in the animal facility at the Universidade Estadual do Norte Fluminense (UENF). Mice indicated for experiments, 8 to 10 weeks old male mice, were transferred to the Animal Biosafety Level 3 facility before infection. This work was carried out according to the recommendations in the Guide for the Care and Use of Laboratory Animals of the UENF. All experimental procedures were approved by the Institutional Animal Care and Use Committee (Permit number 198). Mice were infected intratracheally (i. t.) with 100 CFU/mouse as described previously [10]. The dose of bacteria inoculated in mice was confirmed by a CFU count of lung homogenates at 24 h p.i. and varied ±20% in relation to the dose of infection. To monitor disease progression, mice were weighed before the challenge and then every 7 d. To measure live bacterial burdens in the lung, tissue homogenates were serial diluted and plated on complete Middlebrook 7H10 medium for colony (CFU) count.

### Lung pathology

Left lungs were fixed in 10% buffered formalin and subsequently embedded in paraffin. For histopathological studies, serial 4- to 5-μm sections were stained with hematoxylin and eosin (H&E) to visualize tissue alterations, by Ziehl-Neelsen method to detect the presence of acid-fast bacteria (AFB) and Masson’s trichrome coloration to visualize collagen fibers. For immunostaining, paraffin embedded sections were rehydrated in graded alcohols and exposed to microwave-antigen-retrieval using citrate buffer (10 mM sodium citrate, 0.05% Tween 20, pH 6.0) at 750 W for 15 min. The sections were cooled for 20 min at room temperature. Endogenous peroxidase activity was quenched by the addition of 3% hydrogen peroxide for 5 min. To block nonspecific binding, the sections were incubated in 5% bovine serum albumin for 10 min and treated with a mouse-on-mouse Ig blocking reagent (Vector Lab., CA) for 1 hour at room temperature. The sections were then probed at room temperature for 2 hours with rabbit anti-BCG polyclonal antibodies (Dako, Denmark) in 1:5000 dilution and then with LSAB2- horseradish peroxidase Universal Kit (Dako). Visualization was performed using a diaminobenzidine substrate-chromogen solution (Dako), resulting in a colored precipitate at the antigen site. Sections were counter-stained with hematoxylin. Treatment under these conditions gave negative results with lung sections obtained from uninfected control mice. Additionally, negative control staining of sections from infected mice was performed by replacing the primary antibody with normal rabbit serum or by omitting the primary antibody. The samples were examined with an Axioplan microscope (Carl Zeiss Inc., Germany), and the images of lung sections were captured by Coolpix P995 (Nikon)-coupled device camera.

### Isolation of lung-infiltrating cells

Right lungs were washed with sterile PBS and placed in Petri dishes with RPMI 1640 medium (Gibco, USA). Single-cell suspensions of lung tissue were prepared as described previously [12]. Briefly. the lung tissue was dissected and incubated with digestion medium that contained Collagenase Type IV (Sigma-Aldrich; 0.5 mg/ml) and type IV bovine pancreatic DNAse (Roche Diagnostics; 1 mg/ml) at 37°C for 45 min under agitation conditions. Cells were dispersed with a 10-ml syringe (BD Biosciences) that was fitted with an 18-gauge needle and filtered with a cell strainer (Corning, USA). Red blood cells were depleted with a lysis buffer (0.144 M NH4Cl, 0.0169 M TRIS base, pH 7.4) at 37°C in a 5% CO_2_ atmosphere for 5 min.

Cell viability in the isolated cell suspensions was determined by the trypan blue exclusion test. Numbers of viable (trypan blue-negative) and dead (trypan blue-positive) cells were counted using hemocytometer. An equal numbers of viable cells were taken for flow cytometry analysis and cell culture.

### Phenotypic analysis of lung-infiltrating cells

Cell suspensions obtained from lungs (1x10^6^ cells) were treated with anti-CD16/CD32 mAbs (BioLegend, San Diego, CA) to block Fc receptors and stained using appropriate combinations of antibodies against Ly6G-FITC (clone 1A8), CD11c-PE (clone HL3), Ly6C-APC (clone AL-21) and CD11b-PerCP (all obtained from BD Pharmingen, San Diego, CA). Isotype-matched antibodies, anti-Hamster IgG1-APC, anti-Rat IgG2a-FITC, anti-Rat IgG2b-PerCP and anti-Rat IgG1-PE isotype control (BD Pharmingen, San Diego, CA), were used as controls. Cells were fixed with 2% paraformaldehyde and analyzed by flow cytometry (FACSCallibur, BD Biosciences) using Cell Quest Pro software.

### Cytokine quantification

Lung infiltrating cells (5×10^4^ cells/well) were suspended in complete RPMI 1640 medium (Gibco) with 0.05% gentamicin and 10% fetal calf serum (FCS) and were cultured in 96 well-plates (Corning) at 37°C in a 5% CO_2_ atmosphere for 48 h. Cell culture supernatants were harvested, filter sterilized and stored at −80°C. Levels of IL-1β, IFN-γ, TNF-α, IL-6, IL-17, IL-10, KC, MIP-2, G-CSF and MCP-1 in the cell culture supernatants were measured with a Milliplex multiplex MAP MCYTMAG-70K kit for Mouse Cytokine/Chemokine 96-well plate assay (Millipore Corp.) using Luminex 200™ instrument with xPONENT® software and following to the manufacturer's protocol.

### Statistical analysis

Statistical analysis was performed using Prism4 GraphPad software. To compare multiple groups, one-way analysis of variance (ANOVA) was used, followed by Bonferroni’s multiple-comparison test. Data are shown as mean ± SD. Differences were considered significant where p < 0.05.

## Results

### Kinetics of lung pathology at the acute stage of infection with highly virulent *M*. *tuberculosis* strain M299

**Fig 1:**
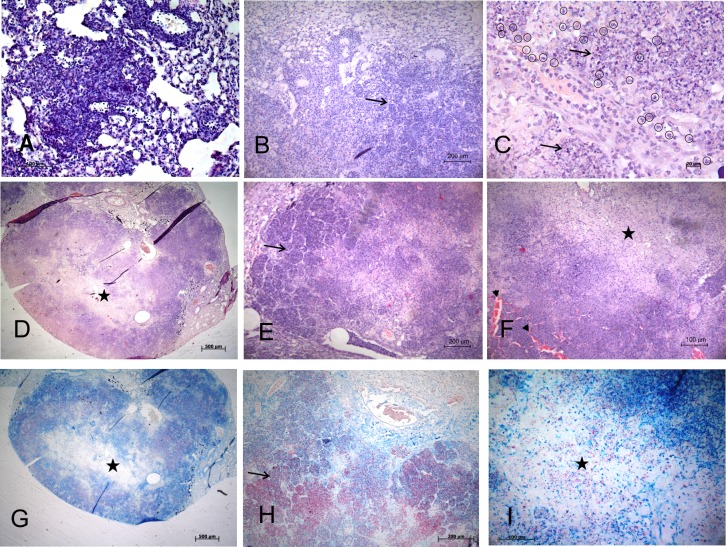
Kinetics of lung histopathology during the acute stage of infection in C57BL/6 mice inoculated with strain M299. Mice were intratracheally infected with ~100 CFU. Pathologic alterations of lung tissue were determined on day 15 p.i. (**A**), day 21 p.i. (**B, C**) and day 28 p.i. (**D- I**) by microscopy of lung sections stained with hematoxylin-eosin (**A- F**) and Ziehl-Neelsen (**G- I**). Panel **A** shows interstitial granulomatous infiltrates on day 15 p.i. Panels **B** and **C** show the development of alveolitis through infiltration of alveoli (black arrows) with inflammatory cells, predominantly by neutrophils (enclosed in black circles). Panels **D** and **G** show coalescing foci of tuberculous pneumonia leading to the formation of tubercules with extensive areas of central caseous necrosis (black stars). The peripheral zone of the necrotic lesion is amplified in **E** and **H**, demonstrating areas of alveolitis (black arrows) with thickened alveolar walls and intra-alveolar cellular exudates composed predominantly of neutrophil debris (**E**) and numerous AFB (**H**). The caseous core is amplified in **F** and **I**. Note the thrombosed septal capillaries (black arrow heads) and obstruction of small airways with cellular debris (white arrow) in the peripheral rim of the necrotic zone (**F**). Numerous extracellular AFB and clumps of bacteria can be seen in the necrotic area (**I**). Data are representative of four independent experiments. Bars represent 500 μm—in the panels D and G, 200 μm—in B, E and H, 100 μm—in A, F and I and 20 μm—in C.

To study the mechanisms leading to pulmonary necrosis in resistant C57BL/6 mice, the mice of this lineage were infected i.t. with a low dose (~ 100 CFU) of the highly virulent strain M299 and the kinetics of histopathological alterations in the lungs were evaluated throughout 28 days of infection. In parallel, mice were infected with the virulent strain H37Rv, known to induce granulomatous infection without necrosis in C57BL/6 mice [10,11], as was also confirmed in this study (**S1 Fig**). Lung pathology induced by strain M299 started as a granulomatous inflammation leading to the formation of multiple granulomas, predominantly around small vessels, which were clearly visible on day 15 after infection (**Fig 1A**). On day 21 p.i., enhanced leukocyte recruitment to the infection foci caused progressive enlargement of primary granulomas. Infiltration of adjacent alveoli with inflammatory cells led to the development of alveolitis, featured by the filling of alveolar spaces with neutrophils and macrophages and thickening of alveolar walls (**Fig 1B**). Distinct stages of neutrophil extravasation and emigration into alveoli were found (**Fig 1C**). On day 28 p.i., enlargement of pneumonia areas was observed, displaying large tubercle formation as a result of the coalescence of primary lesions (**Fig 1D and 1G**). The central area of the tubercles showed evidence of caseous necrosis (**Fig 1F**) with large numbers of extracellular acid-fast bacilli, AFB (**Fig 1I**), surrounded by karyorrhectic masses of degenerating alveolar walls and dying cells. The periphery of the core region was characterized by areas of alveolitis, a retained alveolar structure and large numbers of viable and dying neutrophils and macrophages (**Fig 1E and S2 Fig**) and AFB (**Fig 1H**). Small airways were filled with cellular debris (**Fig 1E and 1F).**

### Bacterial replication in the lungs and C57BL/6 mouse morbidity following infection

To further characterize the severity of disease caused by strain M299 compared with that caused by the laboratory strain H37Rv, we quantified the bacterial burden in the lungs of mice infected by these two strains and measured body weight loss in infected animals as an indicator of morbidity. Additionally, we evaluated clinical signs of a moribund condition, such as lethargy, unresponsiveness, ruffled fur, difficulty walking and/or respiratory difficulty. Mice exhibiting a moribund phenotype and pronounced weight loss (more than 25% of peak body weight throughout the experiment) were euthanized to prevent the suffering and spontaneous death of these animals.

**Fig 2:**
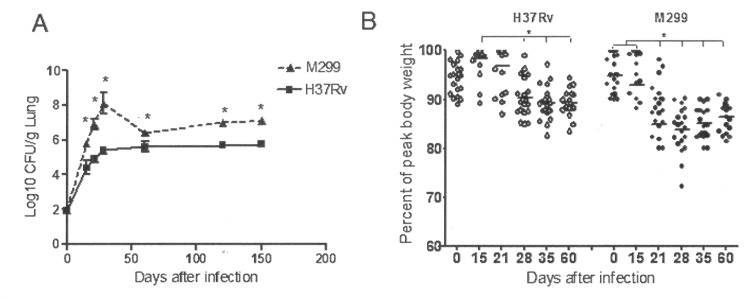
Kinetics of bacterial growth in the lungs and morbidity of C57BL/6 mice inoculated with Mtb strains. Mice were infected i.t. with 100 CFU of the hypervirulent strain M299 and laboratory strain H37Rv. **A.** Bacterial burdens in the lungs were quantified by CFU counting on days 0, 5, 21, 28, 120 and 150 after infection. Data from three experiments (n = 3 for each group and each experiment) are expressed logarithmically as the mean log_10_ CFU standard deviation (SD) (error bars). Mean values that were significantly different from the respective mean value of the group infected by strain H37Rv are indicated by asterisks *, p < 0.05. **B**. Kinetics of post-infection changes in mouse body weight. Weight loss was used as an indicator of morbidity. Data are presented as the percentage of peak body weight of each mice (n = 20, pooled data from two independent experiments). Statistical differences are indicated by asterisks *, p < 0.05.

Following low-dose of infection with strain M299, the bacterial numbers in the lungs were significantly higher than those in mice infected with strain H37Rv at all time points analyzed (**Fig 2A**). CFU numbers increased up to day 28 p.i. in both groups, but the bacterial load in animals infected with strain M299 was almost 2.5 log_10_ greater than in those infected with strain H37Rv. Later on, a significant reduction in the numbers of bacilli was observed in the lungs of mice infected with strain M299 (p < 0.05), but not in those infected with strain H37Rv. However, the resulting lung loads in animals of the former group continued to be at least 1.0 log_10_ higher up to the end of observation period of 150 days. In accordance with the rapid increase in initial bacterial load, mice infected with strain M299 exhibited early morbidity. A significant reduction in the mean body weight in this group of animals was observed as early as day 21 p.i., whereas the weight of animals infected with strain H37Rv dropped only after 28 days of infection and weight loss was less pronounced (**Fig 2B**). In the group infected with strain M299, body weight loss reached peak levels on day 28 p.i. Two animals in this group (10%) with the lowest values of weight (more than 25% body weight loss) exhibited moribund signs and were euthanized. Although animals infected with a low dose of M299 bacilli in most cases survived to at least day 150 p.i. [10], these mice developed severe disease that eventually caused death between 28–35 days after infection, ranging from 0% to 15% in multiple experiments (data not shown).

### Pathogenic mechanisms responsible for the induction of necrosis in the lungs of mice infected with strain M299

**Fig 3:**
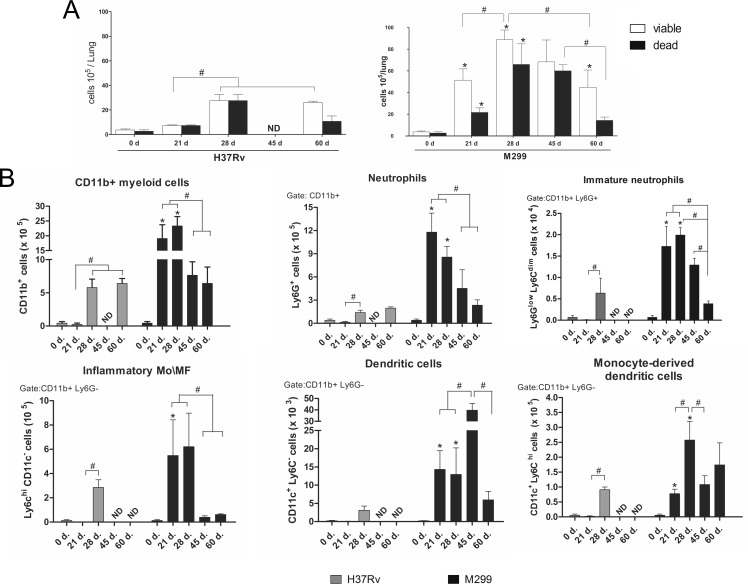
Kinetics of myeloid cell recruitment into the lungs of mice infected with strains M299 and H37Rv. **A**. Total numbers of cells recovered from the lungs over a follow-up period of 60 days after inoculation with bacilli or sterile PBS (control, day 0). Proportions of viable and dead cells were determined by trypan-blue staining. **B**. Myeloid cell populations were identified by flow cytometry. Total numbers of CD11b^+^ myeloid cells per lung and individual myeloid cell subpopulations, including neutrophils (CD11b^+^Ly6G^+^Ly6C^+^cells), immature neutrophil precursors (CD11b^+^Ly6G^low^Ly6C^dim^), inflammatory monocytes and macrophages (CD11b^+^Ly6C^hi^CD11c^-^Ly6G^-^ cells), monocyte-derived dendritic cells (CD11b^+^CD11c^+^Ly6C^hi^Ly6G^-^) and dendritic cells (CD11b^+^CD11c^+^Ly6C^-^Ly6G^-^), were calculated. Values shown are the mean numbers of cells per lung ± SD, n = 8–9 lungs at each time point, pooled data from three independent experiments. Mean values of groups infected by strain M299 that were significantly different from the respective mean value of groups infected by H37Rv strain are indicated by asterisks * (p < 0.05). Significant differences between groups infected by each strain are indicated by lines and octothorpes #, (p < 0.05).

To understand the pathogenic mechanisms leading to distinct disease courses and pathological alterations in the lungs of mice infected with strains M299 and H37Rv, we evaluated the kinetics of leukocyte recruitment into the lungs. To do so, we checked the total numbers and viability of lung-infiltrating cells, as well as the major populations of myeloid leukocytes accumulating in the lungs within 60 d p.i. The total numbers of viable cells recovered from the lungs of mice infected with strain M299 were 10-fold higher than those found in the lungs of H37Rv-infected mice on day 21 p.i. By day 28 p.i., the recruitment of leukocytes into the lungs continued to increase in both groups of infected mice. However, cell numbers in the lungs of mice infected with strain M299 continued to be at least 3-fold higher than those infected with strain H37Rv, although almost half of these cells exhibited signs of necrotic cell death. Later on, lung-infiltrating cell numbers in M299-infected mice started to drop by day 60 p.i., whereas the numbers of cells in the lungs of mice infected with H37Rv bacilli remained unaltered (**Fig 3A**).

To evaluate the composition of inflammatory cells recruited to the lungs after infection, we quantified the main population of myeloid leukocytes by flow cytometry. The gating strategy is shown in (**S3 Fig**).

Myeloid leukocytes were identified as CD11b^+^ cells (**Fig 3B**), and further classified based on their expression of additional markers as neutrophils (CD11b^+^Ly6G^+^Ly6C^+^ cells), inflammatory monocytes\macrophages, Mo/MF (CD11b^+^Ly6C^hi^CD11c^-^Ly6G^-^ cells), dendritic cells, DC (CD11b^+^CD11c^+^Ly6C^-^Ly6G^-^ cells) or monocyte-derived DCs (CD11b^+^CD11c^+^Ly6C^hi^Ly6G^-^ cells).

The kinetics of absolute numbers of myeloid cells recovered from the lungs within 60 d p.i. are presented in **Fig 3B**. The total number of CD11b+ cells in the lungs of mice infected with strain M299 dramatically expanded by day 21 and was more than 10-fold greater in comparison to mice infected with strain H37Rv. Within the following week, large increases in myeloid cell numbers were observed in the lungs of H37Rv-infected mice, whereas in the group of M299-infected mice, this was less pronounced; however, the total numbers of CD11b+ cells continued to be significantly greater in the latter group (~ 4-fold). On day 45 p.i., numbers of myeloid cells in the lungs from M299-infected mice started to drop, whereas in the group of mice infected with strain H37Rv, the cell numbers were maintained at the same level up to day 60. Thus, the most pronounced difference in the numbers of myeloid leukocytes between the groups of mice infected with highly virulent (strain M299) and less virulent (strain H37Rv) bacilli was observed within the first 28 days after infection.

Neutrophils were the predominant myeloid cell population in the lungs of M299-infected animals on day 21, whereas the population of mononuclear phagocytes, including inflammatory Mo/MF and DC, was at least 2-fold less numerous at this time point. By day 28, numbers of viable neutrophils in the lungs of these animals started to drop, whereas the cell numbers of the Mo/MF population, and particularly of Mo-derived DC, continued to increase, leading to a reduction in the proportion of neutrophils in relation to Mo/MF in the lungs. Quantification of the subpopulation of immature neutrophils expressing the Ly6G^low^Ly6C^dim^ phenotype demonstrated a significant increase in the number of these cells in the lungs of mice infected with strain M299 on day 21 p.i., which was maintained at high levels up to day 45 p.i. Numbers of this cell subset in the H37Rv-infected group were at least 3-fold lower than in the former group. CD11b^+^Ly6G^low^Ly6C^dim^cells were demonstrated to express the properties of myeloid suppressors [13], suggesting that these cells can contribute to reducing inflammatory cell recruitment in the lungs of mice infected with strain M299 after 28 days of infection.

In contrast to strain M299, strain H37Rv induced a lower level of myeloid cell recruitment to the lung and the proportions of recruited mononuclear phagocytes were higher than those of neutrophils within the period of observation, confirming the granulomatous character of inflammation induced by this strain.

### Kinetics of lung pathology at the chronic stage of infection with strain M299

**Fig 4:**
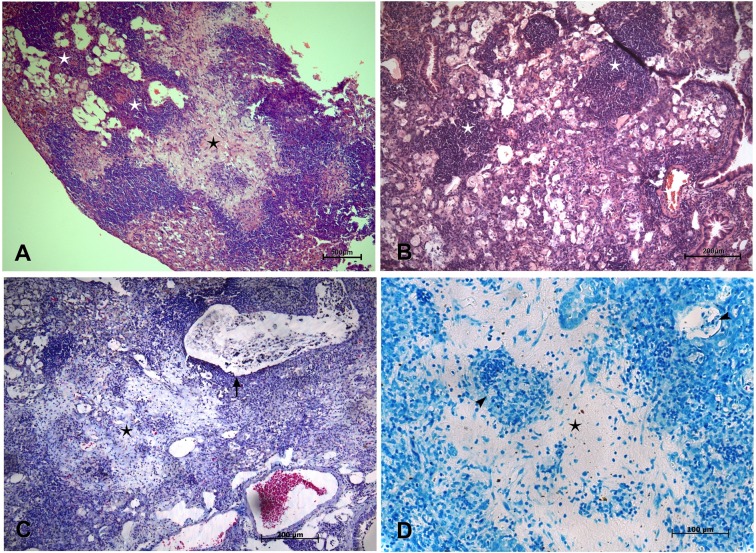
Lung histopathology at the early chronic stage of infection by highly virulent Mtb strain M299. Pathological alterations to lung tissue were observed on day 120 after infection by microscopic analysis of lung sections stained by hematoxylin-eosin (**A** and **B**), Masson´s trichrome (**C**) and Ziehl-Neelsen (**D**). Panel **A** demonstrates a necrotic lesion (black star) partially healed by fibrosis and surrounded by a rim of lymphocytes and lipid-laden foamy macrophages in the external region. Secondary granulomatous lesions are marked by white stars. Panel **B** demonstrates a higher-magnification image of two compact perivascular lymphocyte granulomas, surrounded by foamy macrophages. **C**. Masson’s staining revealed the presence of profuse collagen (in light-blue) in the central area of the necrotic lesion. Note cellular debris in the bronchiole and the absence of a bronchiolar epithelium (black arrow). **D**. Single AFB, exhibiting weak staining (black arrow heads), could be seen in necrotic areas partially healed by fibrosis and in foamy macrophages within alveoli. Bars represent 500 μm- in the panel **A**, 200 μm- in the panels **B** and **C**; and 100 μm- in the panel **D**.

**Fig 5:**
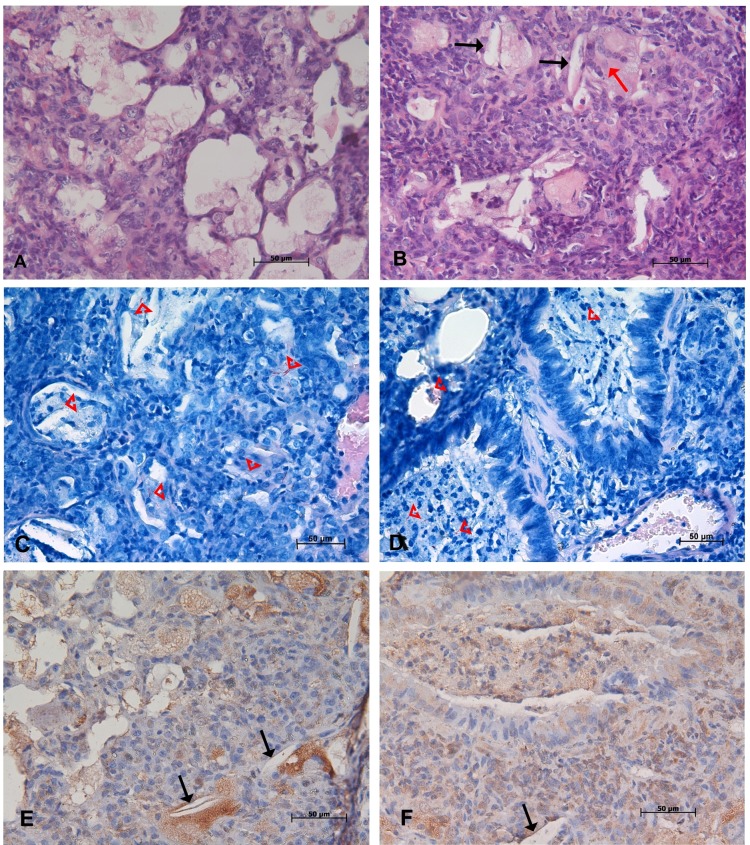
Lung histopathology at the late chronic stage of infection by highly virulent strain M299. Pathological alterations to lung tissue were observed on day 150 after infection by microscopic analysis of lung sections stained by hematoxylin-eosin (**A** and **B**), Ziehl-Neelsen (**C** and **D**) and immunohistochemical staining method for mycobacterial antigens (**E** and **F**). Panels **A** and **B** demonstrate massive intra-alveolar disintegration of foamy macrophages admixed with recruited neutrophils and appearance of multinucleated cells (red arrow, **B**). The resulting cell debris filled small airways (**D** and **F**). Numerous AFB were seen in foamy macrophages and cell debris (red arrowheads, **C** and **D**). Necrotic death of foamy cells led to the liberation of mycobacterial antigens (stained in brown) and the accumulation of needle-shaped cholesterol clefts (black arrows, **B**, **E** and **F**). Bars represent 50 μm.

To evaluate the dynamics of the primary necrotic lesions developing in the lungs of mice infected with strain M299, we monitored lung histopathology over the chronic stage of disease. In our previous study, we established survival curves for C57BL/6 mice inoculated with strain M299, demonstrating that animals infected with a low dose of bacilli exhibited good survival up to 150 days p.i., followed by a gradual decline [10]. Thus, the histopathological alterations in the lungs examined before the day 150 are characteristic of the early chronic stage of lung disease, whereas those observed on day 150 p.i. and later characterized the pathology of the late chronic stage. The data are presented for day 120 (**Fig 4**) and day 150 (**Fig 5**).

The early chronic stage of disease was characterized by gradual expansion of areas dominated by foamy macrophages, whereas compacted secondary granulomas dominated by lymphocytes were scattered (**Fig 4A and 4B**). By day 120 p.i., the primary necrotic lesions were partially healed by fibrosis and were surrounded by a rim of lymphocytes (**Fig 4A**). These lesions presented staining for collagen as demonstrated by the Masson’s trichrome method, thus reflecting scarring brought about by previous necrotic process (**Fig 4C**). Small numbers of weakly stained AFB could be observed within some foamy cells and in the necrotic areas (**Fig 4D**).

Pathological alterations detected on day 150 p.i. differed from earlier observations by a pronounced increase in the degradation of intra-alveolar foamy cells (**Fig 5A and 5B)** that led to the liberation of intracellular bacteria (**Fig 5C and 5D)** and mycobacterial antigens **(Fig 5E and 5F).** The accumulation of neutrophils could be seen in the areas of cell death. Degrading cellular masses, mixed with neutrophils, AFB and mycobacterial antigens, obstructed small airways (**Fig 5D and 5F**). The appearance of multinucleated giant cells can be found in close proximity to numerous cholesterol clefts (**Fig 5B**). In contrast, pathological alterations caused in lungs by the strain H37Rv at this150 day time point p.i. exhibited low level of cell death (**S1 Fig**). Areas dominated by foamy macrophages surrounded compacted lymphocyte granulomas.

### Quantification of cytokines produced by lung-infiltrating cells in the acute and chronic stages of infection

In order to further characterize lung inflammation induced by the hypervirulent strain M299 and the laboratory strain H37Rv, we evaluated the production of pro-inflammatory and anti-inflammatory cytokines by cells isolated from the lungs at different stages of infection (days 0, 21, 28, 45, 60, 120 and 150) and cultured ex vivo.

**Fig 6:**
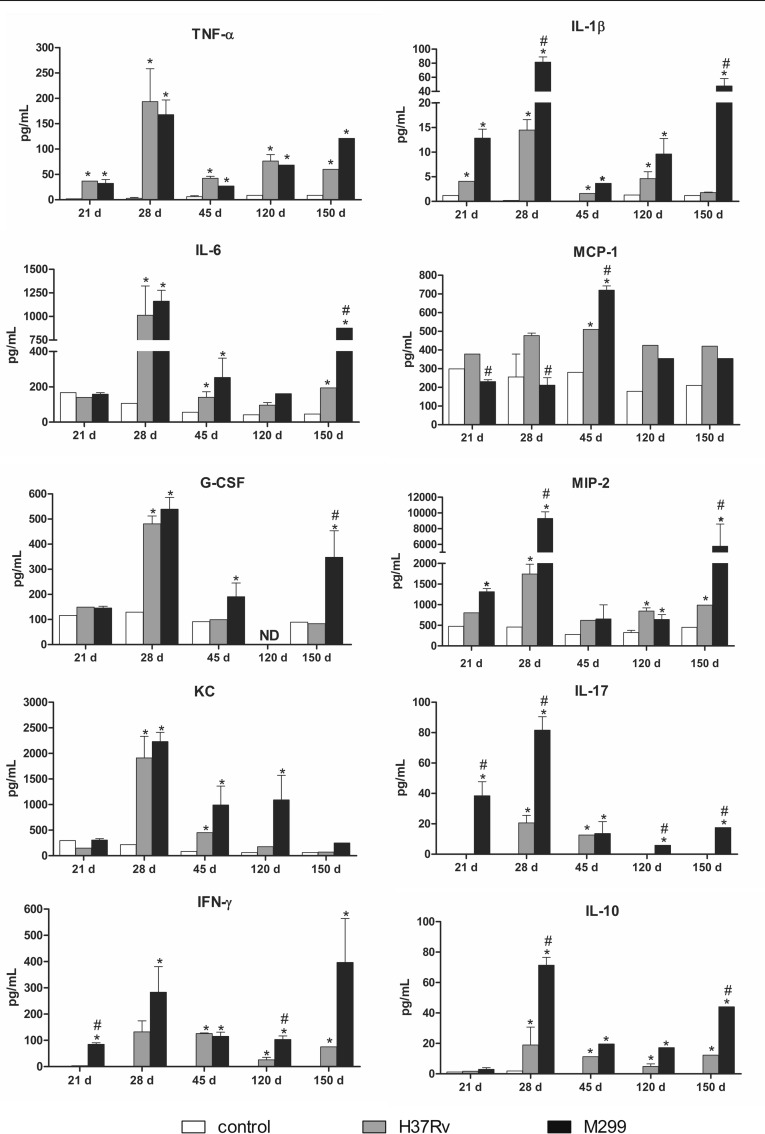
Production of cytokines and chemokines by cells obtained from the lungs of C57BL/6 mice infected with strains M299 and H37Rv. Lung cells were isolated on days 21, 28, 45, 120 and 150 after i.t. infection and cultured in 96-well plates, 5 x 10^4^ cells/well. Culture supernatants were collected after 48 h of incubation and cytokine concentrations were measured by the Multiplex method. The data were obtained in three independent experiments. Significant differences between infected and uninfected groups are indicated by asterisks * (p <0.05). Mean values of groups infected by strain M299 that were significantly different from the respective mean value of groups infected by strain H37Rv are indicated by octothorpes # (p < 0.05). ND- not defined.

At the acute stage of infection, lung-infiltrating cells obtained from mice infected with strain M299 secreted significantly higher levels of pro-inflammatory cytokines (IL-1β), neutrophil-recruiting chemokine (MIP-2/CXCL2) and IL-17, than those infected with strain H37Rv, whereas the secretion of TNF-α, IL-6, G-CSF and KC induced by these strains was similar (**Fig 6**). Both strains stimulated the production of IFN-γ; however, the levels of this cytokine were almost 3-fold higher in cell cultures taken from mice infected with strain M299. The high level of IFN-γ production demonstrated that infection with this strain induced a potent Th1-type immune response, which is important for macrophage activation and the control of Mtb infection. The production of all these cytokines peaked on day 28 (**Fig 6**). In contrast, the secretion of monocyte-recruiting chemokine (MCP-1/CCL2) by cells from mice infected with strain M299 was lower than those infected with strain H37Rv, at least at the acute stage of infection. Induction of MCP-1 by strain M299 significantly increased in the early chronic phase of infection and peaked on day 45 p.i. Production of the inhibitory cytokine IL-10 was rather low on day 21 p.i., but it was increased (18-fold) by day 28. At this time point, lung cells from animals infected with strain M299 produced more than 3-fold higher levels of IL-10 than cells from mice infected with strain H37Rv.

Interestingly, a significant drop in the production of inflammatory cytokines was observed by day 45 p.i. Cytokine inhibition was more pronounced in animals infected with strain M299, demonstrating the capacity of C57BL/6 mice to down regulate inflammation. The level of IFN-γ production was reduced at this time point, coinciding with disease transition to the early chronic phase, characterized by the control of infection and inflammation. Low levels of cytokine production were maintained until day 150 p.i. At this time point, the secretion of some of the pro-inflammatory cytokines (IL-1β, IL-6, G-CSF, MIP-2, IFN-γ), as well as that of anti-inflammatory IL-10, increased again. The increase in cytokine production was observed only in cell cultures obtained from mice infected with strain M299, not in those infected with strain H37Rv. These data suggest the reactivation of lung inflammation in mice infected with strain M299 starting around day 150 after infection (**Fig 6**).

## Discussion

In this study, we investigated the pathogenic mechanisms leading to necrotic lung lesions in a model of pulmonary TB in C57BL/6 mice infected with a highly virulent clinical isolate of Mtb belonging to the modern Beijing sublineage (strain M299) [10].

The results demonstrate that the primary necrotic lesions caused by strain M299 were associated with exacerbated pulmonary inflammation driven by enhanced immigration of myeloid leukocytes. Increased influx of these cells, especially CD11b^+^Ly6G^+^ neutrophils, which represented the largest subpopulation of myeloid cells recruited to the lungs on day 21 p.i., coincided with the development of alveolitis. During the following week, multifocal areas of alveolitis coalesced by day 28 p.i. and formed large tubercles with areas of central acellular necrosis with a caseous core. The development of necrosis coincided with the peak of inflammatory cell infiltration and pro-inflammatory cytokine and chemokine production, whereas the number of neutrophils in the lungs started to decline. Large amounts of intra-alveolar cell debris detected in the areas of alveolitis during the fourth week after infection and the gradual reduction in neutrophil numbers suggested that cellular death was predominantly associated with lung-infiltrating neutrophils. Given the massive neutrophil influx into the airways of C57BL/6 mice in our model, we hypothesized that under these conditions neutrophils may contribute to pathology, although this question deserves further investigation

In murine TB models, the severe necrotic lung pathology associated with an enhanced granulocytic response was seen in genetically susceptible mice, such as Kramnik model based on C3HeB/FeJ mice [14,9], but not in mice of TB-resistant lineages [15]. Our results reveal that employment of a highly virulent Mtb strain is a useful approach to reproduce necrotic lung pathology in the resistant mice. Interestingly, the necrotizing pneumonia induced in C57BL/6 model by strain M299 strongly resembled the Type II lesions (poorly organized neutrophil-driven necrotizing alveolitis), but not the better organized Type I lesions (encapsulated caseous granulomas), occurring in the susceptible C3HeB/FeJ mice after infection with a low dose of reference Mtb strain Erdman [9]. The main difference between animals exhibiting Type II lesions in these two models was that susceptible C3HeB/FeJ mice succumbed to premature death by 28–45 days p.i., whereas the C57BL/6 mice were able to control exacerbated inflammation more efficiently, exhibiting better survival and transition to chronic disease.

Taken together, these data raise questions about the pathogenetic mechanisms that allow some of virulent Mtb strains to cause increased accumulation of neutrophils, hyperinflammation and necrotic alterations in the lungs of TB-resistant mice, and the mechanisms enabling control of the exacerbated inflammation.

Our data highlighting the importance of mycobacterial virulence in the induction of neutrophil accumulation in the lungs are in agreement with recent data of Repasy et al. [16], demonstrating the role of virulence-associated ability of Mtb strains to replicate and induce macrophage necrosis as determinants of neutrophil recruitment in C57BL/6 mice. In our previous studies, we demonstrated that the ability of hypervirulent clinical isolates to induce necrotic cell death in macrophage cultures was significantly higher, when compared with laboratory strains [17,10,11]. Additionally, massive liberation of intracellular danger signals, such as ATP, from dying phagocytes triggers the activation of pro-inflammatory mechanisms contributing to death of lung cells in vivo [18]. Recently, we demonstrated the role of the purinergic P2X7 receptor, a sensor of extracellular ATP, in the promotion of necrotic pulmonary pathology in C57BL/6 mice, presumably through NLRP3 inflammasome-dependent induction of vicious cycles of leukocyte recruitment, cell death and production of pro-inflammatory cytokines, including IL-1β [11]. In accordance with these data, the lung-infiltrating cells of mice infected with strain M299 secreted significantly higher levels of IL-1β, as well as IL-17.

Activation of the inflammasome is now emerging as a critical step in IL-1β-driven inflammation and promotion of Th17 responses, leading to IL-17 production by innate lymphocytes [19]. IL-17 is a cytokine known to increase the production of neutrophil-recruiting chemokines, such as MIP-2, by macrophages and epithelial cells [20,21]. Moreover, excessive IL-17 production during TB might sustain neutrophil recruitment, leading to tissue damage [22]. Accordingly, the production of neutrophil-recruiting cytokines caused by strain M299 in our experiments peaked on day 28 p.i., coinciding with the advanced phase of necrotizing pneumonia associated with massive necrotic death of lung-infiltrating neutrophils, leading to the liberation of a variety of cytotoxic components stored in these cells. Interestingly, large quantities of neutrophil cytosol proteins, such as myeloperoxidase, elastase, MMP-8 and defensins, were detected by a proteomic study in necrotic tissue of caseous granulomas and cavitary lesions, but not in solid granulomas, obtained from patients with severe pulmonary tuberculosis that needed surgical treatment [23]. These data confirm the pathogenic role of neutrophils in necrotic lung pathology, both in humans and animals with severe TB.

In this study, we observed that the decrease in number of neutrophils in the lungs, which started on day 28 after infection with strain M299, coincided with elevated levels of IFN-γ production. In agreement with this observation, previous studies have demonstrated that the death of neutrophils during TB could be induced by the counter-regulatory effects of IFN-γ, limiting neutrophil recruitment and viability in the lungs [24]. IFN-γ is a key negative regulator of IL-17 responses in mycobacterial infections that acts either directly in the differentiation of IL-17-producing cells [25] or indirectly through the induction of anti-inflammatory IDO production by non-hematopoietic cells [26]. Our data suggest that high levels of IFN-γ produced by the lung cells on day 28 p.i. counteracted the proinflammatory effects of IL-17 and promoted neutrophil death.

Another cytokine that was produced in increasing amounts in the lungs of mice infected with strain M299 on day 28 p.i. was IL-10, a cytokine with potent anti-inflammatory and immunosuppressive effects that can be produced by a variety of lymphoid and myeloid suppressor cells. In this study, we showed that the increase in IL-10 production in the lungs coincided with the accumulation of a subpopulation of immature neutrophils expressing the CD11b^+^Ly6G^dim^Ly6C^low^ phenotype, now recognized as lung myeloid-derived suppressor cells (MDSC), with a resemblance to granulocyte-like MDSC [13,27]. The role of these cells in the resolution of bacterial pneumonia through an IL-10-dependent mechanism was demonstrated in a previous study [28]. The role of MDSC in TB is controversial [29]. These cells have been demonstrated to increase predominantly in TB-susceptible murine models developing lung necrosis, such as NOS2^-^/^-^, RAG2^-^/^-^, C3HeB/FeJ [30], I/St [31] or 129S2 mice [29], and were associated with disease progression. Accordingly, ablation of MDSC contributed to the beneficial outcome of primary progressive TB in mice of the TB- susceptible lineages [34]. In resistant C57BL/6 mice infected with a low dose of laboratory Mtb strains, MDSC cells represent a minority of all CD11b^+^ cells [30,29,31]. To our knowledge, the present study demonstrates for the first time that hypervirulent Mtb strains, causing exacerbated inflammation and necrotic lung lesions in C57BL/6 mice, induce the accumulation of MDSC in the lungs, which coincides with the amelioration of lung inflammation and animal survival. These data suggest that, in resistant mice, the accumulation of MDSC cells in the lungs may possibly contribute to the mechanisms limiting inflammation, either directly, through the production of IL-10 and other anti-inflammatory factors, or by triggering regulatory T cells [32] and thereby reducing lung damage. Thus, in resistant C57BL/6 mice that were found to produce higher levels of IFN-γ in response to mycobacterial antigens when compared with susceptible mice [33], the accumulation of MDSC may have a regulatory function in the immune imbalance associated with uncontrolled T cell and macrophage responses, by dampening exacerbated immune activation and reducing collateral damage to the host. In contrast, in susceptible mice, the attempts of host MDSC cells to limit inflammation may further interfere with the weak acquired immune response leading to exacerbation of the infection and death of the host.

The pathologic manifestations observed in C57BL/6 mice at the chronic stage of disease caused by strain M299 largely resembled those that were previously described for mice infected with laboratory Mtb strains [34,15,35]. The disease at this stage was characterized by slow progression associated with a permanent increase in the numbers of intra-alveolar foamy macrophages, bearing persistent bacilli and accumulating large amounts of mycobacterial and host lipids, which resulted in lung consolidation. In the final stages, foamy macrophages started to disintegrate by undergoing necrosis, thus liberating accumulated lipids, bacterial antigens and viable bacteria. These observations led Robert Hunter to hypothesize that pathology of slowly progressive TB in mice resembles the initial stages of post-primary TB in humans characterized by the development of lipid pneumonia before rapidly developing severe inflammation and necrosis [35,36].

The major difference in the chronic disease caused in mice by highly virulent Mtb isolates compared with laboratory strains was the faster progression of lung pathology [15]. Accordingly, chronic infection with Beijing strain M299 resulted in early onset of the advanced disease symptoms reproducing some elements of lipid-like pneumonia, such as necrosis of foamy macrophages, liberation of accumulated lipids and antigens, recruitment of neutrophils and an increase in the production of proinflammatory cytokines by lung-infiltrating cells, starting around day 150 p.i. In our previous study, we demonstrated that a large proportion of animals infected with strain M299 died between day 150 and day 210 p.i. [10], suggesting that the observed aggravation of lung inflammation may contribute to host mortality.

In conclusion, this model of TB in C57BL/6 mice infected with the highly virulent *M*. *tuberculosis* strain M299 reliably reproduces the hyperinflammatory response of a TB-resistant immunocompetent host to highly virulent mycobacteria. This response was strongly associated with excessive recruitment of polymorphonuclear and mononuclear phagocytes and proinflammatory cytokine production in the lungs that can contribute to pulmonary necrosis. Taking into consideration that hyperinflammation is a common feature of severe TB, irrespective of factors causing an unfavorable course of the disease (genetic susceptibility of the host, immune suppression, increased virulence of the bacteria, etc.), the proposed model may be useful for testing of new approaches for the treatment of severe TB, such as host-directed adjuvant therapy aimed at reducing the hyperinflammatory response, the suppression of neutrophil recruitment to the lung and the prevention or reduction of pulmonary necrosis, all of which may be used to augment conventional anti-mycobacterial therapeutic regimens used for TB treatment.

## Supporting information

S1 Fig Lung histopathology in C57BL/6 mice following infection by Mtb strain H37Rv. Mice were intratracheally infected with ~100 CFU. Pathologic alterations of lung tissue were determined on day 28 p.i. (**A,B,C**) and day 150 p.i. (**D,E,F**) by microscopy of lung sections stained with hematoxylin-eosin (**A,B,D,E**) and Ziehl-Neelsen (**C,F**). Panel **A** shows small perivascular and peribronchiolar granulomatous infiltrates on day 28 p.i. with inset showing one of this infiltrates magnified (**B**). Only a few, if any, AFB can be seen in primary granulomas (**C**). Progression of lung pathology by 150 d p.i (**D**), with inset demonstrating secondary lymphocyte granulomas surrounded by foamy macrophages confined within the alveolar spaces (**E**). Few AFB (black arrows) are found in foamy macrophages (**F**). Scale bars: 1000 μm in (**A**), 500 μm in (**D**), 200 μm in (**E**) and 50 μm in (**B,C,F**).
(TIF)

S2 Fig Neutrophil extravasation and emigration into the alveolar spaces on day 28 after infection of mice with Mtb strain M299. Different stages of neutrophil extravasation and translocation into lung tissue are demonstrated in panels **A, B** and **D** (HE staining) and **C** (ZN staining). Images **A** and **C** are serial sections of lesions. The neutrophils are denoted by black circles. Alveolar spaces are filled predominantly by neutrophils (red arrows), macrophages (black arrows), foamy cells (black arrowheads) and large amount of cellular debris (**A, B** and **C**). Numerous intracellular and extracellular AFB (red bacilli) can be seen in panel **C**. Small airways are filled with leukocytes and cell debris **(D**). Scale bars: 50 μm.
(TIF)

S3 Fig Gate strategy to analyze subpopulations of myeloid cells in the lungs. Myeloid populations were identified as CD11b^+^ cells (gate R2) and further classified based on their expression of Ly6G marker, as Ly6G^+^ (gate R3) or Ly6G^-^ (gate R4). The Ly6G^+^ cells were further discriminated by a Ly6C marker as follows: neutrophils (Ly6C^+^Ly6G^+^, gate R11) and less mature neutrophil precursors, corresponding to G-MDSC (Ly6C^dim^Ly6G^low^, gate R8 and R9). The Ly6G^-^ cells were classified by the Ly6C and CD11c markers as inflammatory monocytes/macrophages (Ly6C^hi^ CD11c^-^, gate R5); DCs (Ly6C^-^ CD11c^+^, gate R6) and monocyte-derived DCs (Ly6C^hi^CD11c^+^, gate R7).
(TIF)
