# Correction: Hypervirulent *Mycobacterium tuberculosis* strain triggers necrotic lung pathology associated with enhanced recruitment of neutrophils in resistant C57BL/6 mice

**DOI:** 10.1371/journal.pone.0175652

**Published:** 2017-04-06

**Authors:** Fabrício M. Almeida, Thatiana L. B. Ventura, Eduardo P. Amaral, Simone C. M. Ribeiro, Sanderson D. Calixto, Marcelle R. Manhães, Andreza L. Rezende, Giliane S. Souza, Igor S. de Carvalho, Elisangela C. Silva, Juliana Azevedo da Silva, Eulógio C. Q. Carvalho, Afranio L. Kritski, Elena B. Lasunskaia

The eighth author’s name is spelled incorrectly. The correct name is: Giliane S. Souza.
