# Supplementary figures and images for: Hypervirulent Mycobacterium tuberculosis strain triggers necrotic lung pathology associated with enhanced recruitment of neutrophils in resistant C57BL/6 mice
Source: PLoS One. 2017 Mar 17;12(3):e0173715. doi: 10.1371/journal.pone.0173715 (PMC5357019; doi:10.1371/journal.pone.0173715)

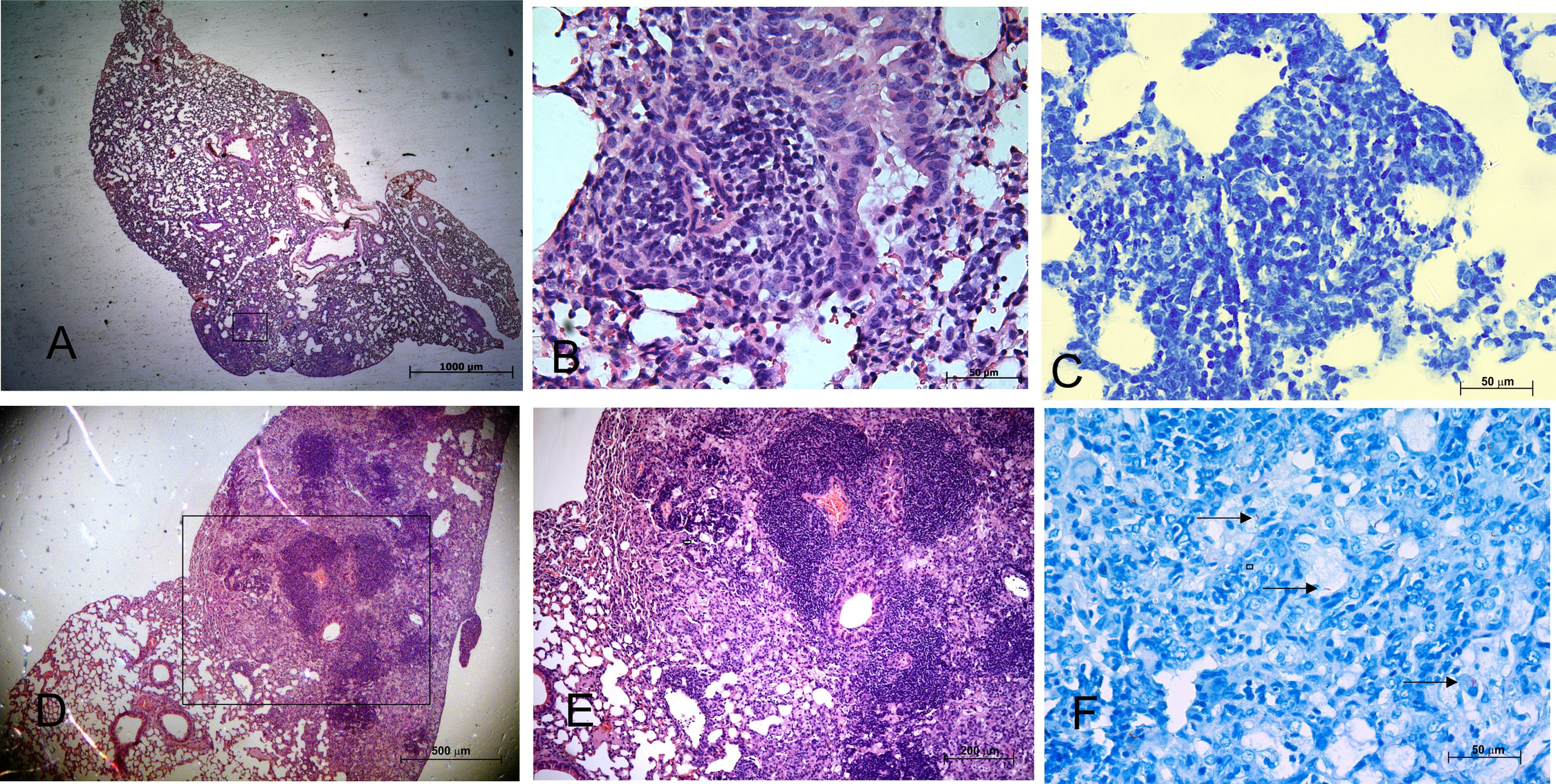

Supplement: S1 Fig — Mice were intratracheally infected with ~100 CFU. Pathologic alterations of lung tissue were determined on day 28 p.i. (A,B,C) and day 150 p.i. (D,E,F) by microscopy of lung sections stained with hematoxylin-eosin (A,B,D,E) and Ziehl-Neelsen (C,F). Panel A shows small perivascular and peribronchiolar granulomatous infiltrates on day 28 p.i. with inset showing one of this infiltrates magnified (B). Only a few, if any, AFB can be seen in primary granulomas (C). Progression of lung pathology by 150 d p.i (D), with inset demonstrating secondary lymphocyte granulomas surrounded by foamy macrophages confined within the alveolar spaces (E). Few AFB (black arrows) are found in foamy macrophages (F). Scale bars: 1000 μm in (A), 500 μm in (D), 200 μm in (E) and 50 μm in (B,C,F). (TIF) [file pone.0173715.s001.tif]

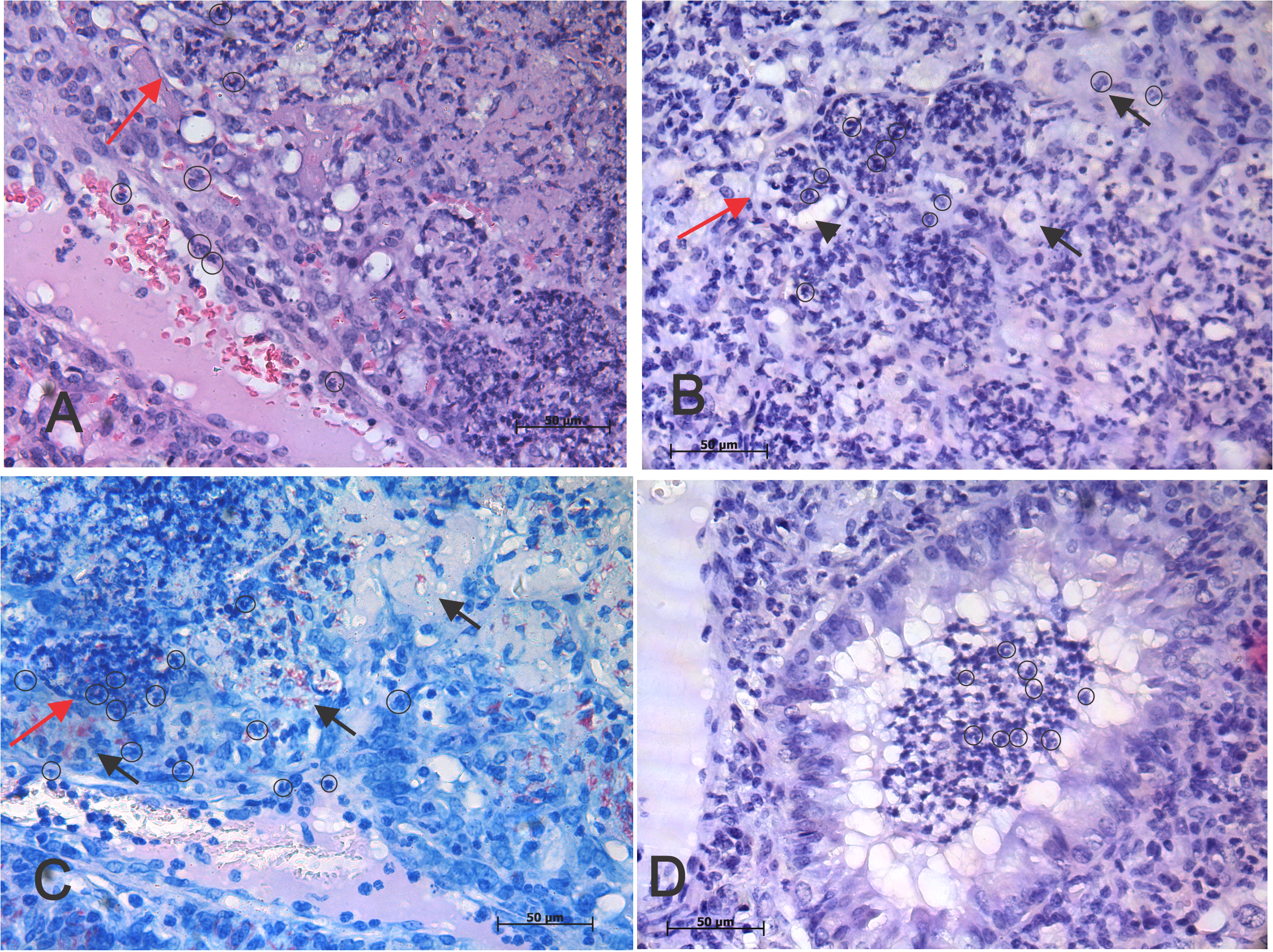

Supplement: S2 Fig — Different stages of neutrophil extravasation and translocation into lung tissue are demonstrated in panels A, B and D (HE staining) and C (ZN staining). Images A and C are serial sections of lesions. The neutrophils are denoted by black circles. Alveolar spaces are filled predominantly by neutrophils (red arrows), macrophages (black arrows), foamy cells (black arrowheads) and large amount of cellular debris (A, B and C). Numerous intracellular and extracellular AFB (red bacilli) can be seen in panel C. Small airways are filled with leukocytes and cell debris (D). Scale bars: 50 μm. (TIF) [file pone.0173715.s002.tif]

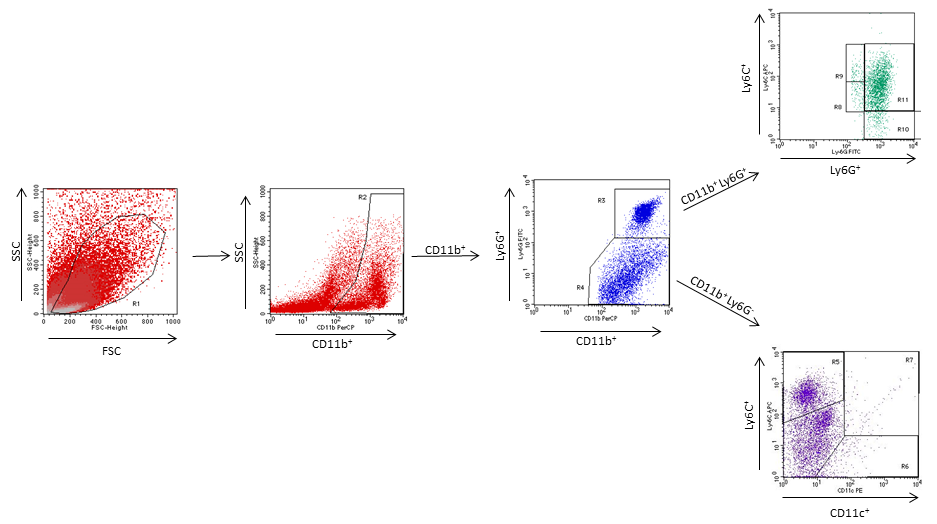

Supplement: S3 Fig — Myeloid populations were identified as CD11b+ cells (gate R2) and further classified based on their expression of Ly6G marker, as Ly6G+ (gate R3) or Ly6G- (gate R4). The Ly6G+ cells were further discriminated by a Ly6C marker as follows: neutrophils (Ly6C+Ly6G+, gate R11) and less mature neutrophil precursors, corresponding to G-MDSC (Ly6CdimLy6Glow, gate R8 and R9). The Ly6G- cells were classified by the Ly6C and CD11c markers as inflammatory monocytes/macrophages (Ly6Chi CD11c-, gate R5); DCs (Ly6C- CD11c+, gate R6) and monocyte-derived DCs (Ly6ChiCD11c+, gate R7). (TIF) [file pone.0173715.s003.tif]
